# Prediction of Work from Home and Musculoskeletal Discomfort: An Investigation of Ergonomic Factors in Work Arrangements and Home Workstation Setups Using the COVID-19 Experience

**DOI:** 10.3390/ijerph20043050

**Published:** 2023-02-09

**Authors:** Justine M. Y. Chim, Tien Li Chen

**Affiliations:** 1 College of Design, National Taipei University of Technology, Taipei 10608, Taiwan; 2 Chim’s Ergonomics and Safety Limited, Hong Kong SAR, China

**Keywords:** COVID-19, work from home, musculoskeletal health, home workstation, work arrangements, office worker, home furniture, work chair, laptop computer

## Abstract

The COVID-19 pandemic provided an opportunity for office workers to experience work from home (WFH). The aims of this study are to investigate the prevalence rate of musculoskeletal discomfort (MSD) and the work conditions of homeworkers during WFH as well as to evaluate the association and predicted risk of ergonomic factors and MSD. A total of 232 homeworkers completed questionnaires. Chi-square test and logistic regression were used to analyze the association and prediction of work arrangements and home workstation setups and musculoskeletal outcomes. The result showed that 61.2% of homeworkers reported MSD while WFH. Because of the small living spaces in Hong Kong, 51% and 24.6% of homeworkers worked in living/dining areas and bedrooms, respectively, potentially affecting their work and personal life. Additionally, homeworkers adopted a flexible work style, but prolonged computer use while WFH. Homeworkers who used a chair without a backrest or a sofa could predict a significantly higher risk of MSD. The use of a laptop monitor posed about a 2 to 3 times higher risk of suffering from neck, upper back, and lower back discomfort than the use of a desktop monitor. These results provide valuable information to help regulators, employers, homeworkers, and designers create better WFH guidelines, work arrangements, and home settings.

## 1. Introduction

Remote work is not a new concept that emerged from the pandemic the novel coronavirus disease (COVID-19) caused. Dyer and Shepherd [1] claimed to have had experience running remote companies for 30 years. Nilles [2] coined the word ‘telecommuting’ quite early in 1975. In the literature, telecommuting is also called work-from-home (WFH), telework, remote work, electronic work, and virtual work [3,4]. Part-time homeworkers are those who work some days at home and some days at the office during a normal work week [5]. The initial intentions of telework arrangements before the COVID-19 pandemic were as follows: to help ensure a work-life balance [6,7,8], to reduce real estate costs [7,9], and to reduce air pollution and traffic congestion [7,10]. 

Traditionally, employees work from an office, which is the main place to perform their work duties. The home, meanwhile, is a place where one carries out personal and family activities. The images of the workplace and the home are those of two separate environments: that of the employers and that of the employees. Suitable furniture and sufficient equipment are necessary to carry out work activities at home. Many employees were not ready for a home office setting prior to the COVID-19 pandemic. Employees found it difficult to separate work activities from home activities and balance their work, and family lives during the forced WFH arrangements the pandemic necessitated [11,12,13]. Employees were also uncertain about future work arrangements after new work practices were implemented. Employees and companies agreed that the former was more productive during WFH, but many employees still preferred to go to the office than do remote work [1]. 

Some discussions have been held on the implementation of telework in the countries of the European Union. Some of these countries have developed hard laws and collective agreements, such as Poland, Germany, and Belgium. Hard laws refer to regulations, directives, and decisions that regulate employees’ right to remuneration and fair working conditions, as well as employers’ obligations to teleworkers, whereas soft laws refer to guidelines and opinions on telework arrangements. Collective bargaining between trade unions and employer organizations on telework has been implemented at the national, cross-industry, or company level [10,14]. 

Recent studies reported positive and negative outcomes of WFH in different home working arrangements and home conditions [15,16,17,18]. On the positive side, at the personal level, studies reported lower time pressure [19], better control of work schedules [17], higher work motivation [20], higher life satisfaction [21], and less work-family conflict [19]. At the company level, teleworkers achieved higher work engagement [22] and higher organizational performance [20]. On the negative side, teleworkers perceived lower subjective well-being [18,23], lower psychological well-being [24], negative relationships with co-workers [17], and conflict within the organization [25]. Teleworker stress level results have been controversial because some teleworkers reported higher stress levels [16,18,23,24,25], whereas others reported lower stress levels [22,26]. 

### 1.1. COVID and WFH in Hong Kong

The current study was conducted in Hong Kong during the fifth wave of the COVID-19 pandemic in 2022. Hong Kong has more than seven million people and is densely populated. After the rollback of social contact measures, the Hong Kong government published WFH guidelines for government employees and sent advisory notices to encourage private employers to follow WFH arrangements [13,27]. There has been little discussion in the literature on the implementation of WFH arrangements in Hong Kong, except for Leung [28] in 2004 and Vyas and Butakhieo [13] in 2021.

The WFH environment in Hong Kong was generally found to be inadequate during the COVID-19 pandemic. The shortage of housing in Hong Kong has forced most families to live in tiny flats or rooms [29]. These inadequate living spaces present a real challenge for WFH. Workspaces are insufficient to accommodate all family members. A study discussing the average living space per person in Hong Kong, Tokyo, Japan, and Singapore in 2018 found that Hong Kong had the smallest living space per person, only 161 square feet. This was approximately 25% lower than Tokyo and 60% lower than Singapore [13,30].

A study concluded that, currently, WFH is not one of the preferred solutions for most Hong Kong employees. The study highlighted the issues with WFH in Hong Kong, such as the lack of adequate workspaces, information, and communication technology resources, and access to business documents during remote work [13]. Studies of work arrangements and home workstation setups conditions, as well as the prevalence rate of work-related musculoskeletal symptoms during WFH in Hong Kong, are lacking.

The Occupational Safety and Health (Display Screen Equipment) (DSE) Regulation (Cap. 509B) was enacted in 2003 in Hong Kong. The regulation has six provisions: (1) workstation risk assessment, (2) risk assessment record, (3) reduction in risks, (4) workstation requirements, (5) health and safety training, and (6) users’ compliance with a safe system of work. It aims to protect the health and safety of employees who use DSE at work for a prolonged period [31]. In principle, the same regulation applies to homeworkers if their employers require them to work with a computer at home.

### 1.2. Musculoskeletal Health of Prolonged Computer Users

The neck, lower back, and shoulders are the three most commonly reported body regions that suffer from musculoskeletal symptoms among computer users [32,33,34,35]. For example, in a Hong Kong study, 61.9% (N = 1085) of office workers reported two or more instances of physical discomfort related to computer work. Further, they reported the shoulder (71%), neck (49.5%), and lower back (39.4%) as the three main regions of discomfort. The prevalence rate of musculoskeletal discomfort (MSD) among office workers in Hong Kong is alarming [36]. 

Other studies also highlighted the relationship between computer use and musculoskeletal disorders. Many studies reported high prevalence rates of MSD among office workers. Risk factors for musculoskeletal disorders were identified, including psychosocial, organizational, and physical elements in the workplace setting, such as computer usage time, working posture and movement, workplace conditions, repetitiveness, rest breaks, demographic characteristics, and computer user characteristics [32,34,37,38].

### 1.3. Work Arrangements and MSD

Ardahan and Simsek [39] confirmed that office workers who use computers for more than seven hours a day report a higher rate of musculoskeletal symptoms in all regions of the body. Similarly, the severity of musculoskeletal pain in many body regions is positively correlated with prolonged daily use of computers. Office employees are exposed to greater musculoskeletal risks with more static postures and increasing computer use times [34].

A study covering the 2015 to 2019 period concluded that more than 96% of 1701 office employees in Hong Kong spent at least six hours a day on computing operations. Meanwhile, a 2002 study found that 48% of 368 office employees spent at least four hours a day on a computer [35,40]. These results indicate that office workers in Hong Kong are spending longer hours on computers, with the duration of prolonged computer usage doubling over the last two decades. 

### 1.4. Home Workstation and MSD

Suitable furniture is essential to create a conducive work environment. Additionally, working postures should be adapted according to the design and condition of the workstation. These factors area ultimately associated with the physical well-being and musculoskeletal health of the employee [41,42]. Ergonomic furniture can reduce MSD or visual discomfort when sitting and working at a workstation. Furniture selection becomes fundamental for workstation setup and the adoption of healthy postures [42,43,44,45,46].

A workstation should be ergonomically designed to allow users to adopt a safe and healthy working posture. In a workstation setup, the following are important: screen quality and position, keyboard and mouse design, support for forearms, work surface size, desk height, and foot support, chair back support and adjustability, body size of the user, illumination, noise management, ventilation, and air quality [31,47,48]. 

Home office ergonomics should be promoted so that homeworkers know how to set up workstations. Government bodies have published guidelines and reference information on WFH setups for creating a healthy home office environment [49] and have promoted ergonomic principles for office furniture and workstations in office settings [31,47,48,50].

### 1.5. Purpose of the Study

The purposes of the study were (1) to investigate the prevalence rate of musculoskeletal problems in computer-based homeworkers during WFH in Hong Kong, (2) to identify work arrangements and conditions of computer-based homeworkers during WFH, and (3) to evaluate the association between work arrangements and home workstation setups and the predicted risk of MSD of computer-based homeworkers during WFH. 

## 2. Materials and Methods

### 2.1. Research Design and Data Collection

A cross-sectional study was conducted measuring the outcomes and exposures of study participants at one point in time. The target population was office workers who were required to use computers for work almost every day and who were required to WFH during the COVID-19 outbreak in Hong Kong. The study was conducted using a structured online questionnaire on an online survey platform. Data were collected using the random sampling method. The first page of the questionnaire highlighted the confidentiality statement, which aimed to protect the privacy of all participants. The collected data were anonymized. Participants were presumed to have completed the questionnaire voluntarily and to have given informed consent. 

The bilingual (English and Chinese) questionnaire was divided into four parts with questions on the type of home workstation, musculoskeletal health, work arrangements during WFH, and demographic information. Convenience sampling was used to access members of the population at a low cost during the COVID-19 pandemic. Emails, messages, and posts were sent to 10 Hong Kong companies and five academic societies inviting participants. Three corporations and three academic societies agreed to distribute the invitations to their members. Additionally, invitation messages and posts with links to the questionnaire were released on social networks. Participants completed the online questionnaire voluntarily. The completed questionnaires were collected between 16 May 2022 and 15 June 2022 during the fifth wave of the COVID-19 pandemic in Hong Kong. Respondents were offered the opportunity to receive supermarket vouchers in a draw if they completed the questionnaires and provided their contact details.

### 2.2. Measurement

In this study, (a) demographic characteristics, (b) work arrangements, (c) home workstation setups, and (d) MSD during WFH data were collected.

(a) Demographic Characteristics

Demographic data included gender, age, employment status, and employment position in the company.

(b) Work Arrangements

Work arrangements data included the average number of days of WFH in a week, work hour changes, total hours of work on a computer on a workday, regular meal breaks, and rest break arrangements.

(c) Home Workstation Setups

Survey participants selected the workspace, furniture, and equipment type that was most representative of their WFH period during the fifth wave of the COVID-19 pandemic. Measures of home workstation setups covered five areas: workspace, desk, chair, display screen, and input devices. Based on the guidelines and the literature on the most ergonomic to the least ergonomic designs and arrangements, during the data analysis, each area was classified into two to three groups [31,47,48,51]. The sample question on the workspace arrangement was ‘Workspace is mostly arranged at …’ The options were ‘living area,’ ‘dining area,’ ‘spare room/study room or reading area,’ ‘bedroom,’ ‘kitchen and other.’

(d) MSD

MSD was assessed by adopting the improved version of the MSD assessment tool, which Marley and Kumar [52] developed and validated. The self-report assessment tool consisted of ratings on the frequency of discomfort ranging from 1 to 3 (0 = never, 1 = rarely [few times/month], 2 = frequently [few times/week], 3 = constantly [nearly every day]) and ratings on discomfort ranging from 0 to 10 (0 = no discomfort, 2 = fairly uncomfortable, 5 = moderate discomfort, 8 = very uncomfortable and 10 = extreme discomfort) for 14 body parts including the eyes. The MSD assessment tool is a reliable tool to indicate the risk of musculoskeletal injury [52,53]. The likelihood of an employee seeking medical treatment for MSD from the assessment tool results was not included in the study objective.

### 2.3. Statistical Analysis

Statistical analysis includes descriptive statistics and inferential statistics. Demographic characteristics were used to understand the individual profiles of the surveyed homeworkers. Homeworkers’ work arrangements, including the number of WFH months, the number of WFH days per week, working hours during WFH, hours of computing work, and meal and rest break patterns, were presented as descriptive statistics.

The home workstation setups in five areas, workspace, work desk, chair, display screen, and input devices, were summarized. Further, the prevalence of work-related MSD with discomfort level details in 14 body regions per homeworker was reported in the descriptive statistics sections. 

In order to evaluate the association between work arrangements, home workstations, and MSD of computer-based homeworkers during WFH, inferential statistical analysis was conducted. 

First, univariate analysis was conducted using the chi-square test for each independent variable in demographic characteristics, work arrangements, and home workstation setup factors. The aim was to compare the differences between no discomfort and discomfort in any body region, neck/lower back/right shoulder region, and individual body regions (e.g., neck, lower back, upper back, and right shoulder). Second, to reduce the number of variables in the logistic regression analysis, variables that reached the significance level of *p* < 0.05 in the chi-square test were retained for the multivariate regression model. In the logistic regression analysis, the reference group was carefully selected after considering the past literature. The first group was the reference category in the analysis [54]. Finally, a *p*-value < 0.05 was considered statistically significant for the prediction of MSD. All data were analyzed using IBM SPSS Statistics (version 29).

## 3. Results

Data were collected from 16 May 2022 to 15 June 2022. A total of 237 surveys were submitted online. Out of them, 232 surveys were considered valid. The criteria for validity were that the respondents were office workers, had experience with WFH during COVID-19, and had completed the entire questionnaire. Respondents who reported using a mobile phone for working at home and who were not required to work with a computer almost every day were excluded. 

Because of the significant decrease in COVID-19 reported cases in the fifth wave since early May 2022, the WFH arrangement also gradually changes. Thus, the research team decided to complete the data collection in a month after considering the acceptable response rate.

### 3.1. Demographic Characteristics of Homeworkers

Table A1 provides the demographic characteristics of homeworkers. Among the 232 valid responses, the majority were women (69%) in the age group of 45 to 54 (39.2%) and 35 to 44 (33.2%) years. These were the two main age groups in the study. The body height of the homeworkers was determined through the questionnaire. The highest percentage of homeworkers (25%) was 156 to 160 cm tall. Regarding their employment status, 94% of them worked full-time, and 32.3% and 30.2% of them were general staff and middle management workers, respectively.

### 3.2. Prevalence Rate of Work-Related MSD

#### MSD Level

A total of 61.2% of homeworkers self-reported discomfort for at least one body region, and 38.8% reported no discomfort. The results of the level of self-reported discomfort for 14 body regions showed that, except for the neck, the percentage of respondents with no discomfort was higher than that with discomfort (Table 1). For the neck, 50.4% of participants reported discomfort. For the neck (50.4%), the upper back (45.7%), the lower back (44%), and the right shoulder (41.4%), more than 40% of participants reported discomfort. The top three mean discomfort scores out of 10 were for the neck (2.31), lower back (1.97), and right shoulder (1.87); the score range was about 2, which was fairly uncomfortable. Left lower leg (0.60) was reported to have the lowest mean level of discomfort. A high percentage of homeworkers (42.7%) reported eye discomfort while working from home.

Table 2 shows the frequency of discomfort for three regions of the body: neck, lower back, and right shoulder. These three had the highest mean scores for the level of discomfort. Never any discomfort and rarely any discomfort (i.e., a few times/month) were the most reported discomfort frequencies for the neck, lower back, and right shoulder. A total of 11 to 12% of homeworkers reported suffering from the neck (11.6%), lower back (12.1%), or right shoulder (11.2%) discomfort nearly every day. 

Body regions with MSD on the right side were reported more frequently than those on the left side. For example, participants reported 9% higher MSD for the wrist and 4.8% higher MSD for the shoulder on the right side. The right shoulder was also the third-highest reported body region for MSD. These results showed the dominant musculoskeletal issue on the right side of the body.

### 3.3. Work Arrangements of Homeworkers

At the time of the study, during the fifth COVID-19 wave in Hong Kong, more than 30% of the surveyed homeworkers had already worked from home for two to three months since 1 January 2022. About 40% of them were asked to work from home five days or more per week. Regarding working hours at home, 41.4% of homeworkers reported that working hours in the office and at home were almost the same, whereas 17.2% reported increased working hours at home. Most of the respondents (44.8%) reported working eight hours or more on their computers at home. The second highest group (34.5%) reported working six to eight hours. Regarding rest break arrangements, more than 50% of respondents ‘often’ or ‘always’ scheduled regular meal breaks during WFH, whereas most respondents (38.4%) did not schedule rest breaks (Table 3).

### 3.4. Home Workstation Setups for Homeworkers

Regarding the setup of the workstation, most homeworkers worked in the living area (32.8%) and at the dining table (40.9%) and used the dining chair (34.1%) during WFH. The bedroom (24.6%) and the dining area (22.4%) were the second and third-highest reported workspaces. About 20% of homeworkers did not use a proper chair for WFH. They used a bench without back support, a folding chair without back support, a sofa/armchair, or a lower chair/kid-sized chair/bar tool. A total of 4.3% of participants worked on the floor/on the bed. 

More than 60% of homeworkers only used a laptop screen as a display screen. Comparatively, only 12.9% (9.9% and 3.0%) of homeworkers used desktop monitors as display screens. A total of 50% used laptop keyboards and an external mouse as input devices. The second highest number of participants used an external keyboard and mouse (28%) during WFH (Table 4).

### 3.5. Factors Associated with MSD

#### 3.5.1. Demographic Characteristics

The chi-square test was used to find significant differences in self-reported MSD between groups in the bivariate analysis. A *p*-value of 0.05 was used for statistical comparisons. Gender was significantly associated with MSD. Gender and body height were significantly associated with discomfort reported at the neck/lower back/right shoulder region(s). Table A2 and Table A3 present the results of the chi-square test.

#### 3.5.2. Work Arrangements

For work arrangements with six factors, including the number of WFH months, the number of WFH days per week, working hours during WFH, hours with computing work, regular meal breaks, and rest break patterns, only the arrangement of regular meal breaks was associated with MSD and neck/lower back/right shoulder discomfort (Table A4 and Table A5).

#### 3.5.3. Home Workstation Setups

For home workstation setups with five ergonomic factors, the type of work desk, chair, and display screen used was associated with MSD (Table A6). In the analysis of individual body regions, including the neck, lower back, and right shoulder, the type of chair used was associated with neck, lower back, and right shoulder discomfort, and the type of display screen used was associated with neck discomfort and upper back (Table A7).

#### 3.5.4. Summary of Factors Associated with MSD

Table 5 and Table 6 present the summarised results of a bivariate analysis of the factors associated with MSD. There were three, six, and five independent factors of demographic characteristics, work arrangements, and home workstation setups, respectively. 

### 3.6. Predictors of MSD by Multivariate Logistic Regression Analysis

Table 7, Table 8 and Table 9 present the findings of logistic regression models for predictors among anybody region, neck/lower back/right shoulder, and individual body region in demographic characteristics, work arrangements, and home workstation setups. 

The *p*-value < 0.05 was set to determine the statistical significance of the variable in the logistic regression model. The odds ratio (OR) shows the strength of the association between two variables. An OR greater than one represents the risk of exposure to MSD associated with an increased likelihood of MSD occurring [54]. 

#### 3.6.1. Demographic Characteristics

Logistic regression was performed to determine the effects of gender and body height on the probability that participants had MSD and neck/lower back/right shoulder discomfort. Table 7 shows the *p*-value, OR, and 95% confidence interval (CI) (lower to upper bounds). 

Gender was a statistically significant predictor of MSD (*p* = 0.015, OR = 2.330, 95% CI = 1.181–4.597) and neck/lower back/right shoulder discomfort (*p* = 0.016, OR = 2.280, 95% CI = 1.164–4.467). Female homeworkers showed a higher risk of developing MSD and neck/lower back/shoulder MSD than male homeworkers. The OR for gender was greater than one, which means that the risk of females suffering from MSD and neck/lower back/right shoulder discomfort was 2.330 times and 2.280 times higher than for males, respectively. Body height was a statistically insignificant predictor of MSD and neck/lower back/right shoulder discomfort. Logistic regression models were statistically significant for MSD (χ^2^(2) = 9.679, *p* < 0.05) and neck/lower back/right shoulder (χ^2^(2) = 10.73, *p* < 0.05) models.

#### 3.6.2. Work Arrangements

Logistic regression was performed to determine the effects of work arrangements on taking breaks and on the probability that participants had MSD and neck/lower back/right shoulder discomfort. Table 7 shows the *p*-value, OR, and 95% CI (lower to upper bounds). 

Regular meal breaks were a statistically significant predictor of MSD (*p* = 0.024, OR = 2.165, 95% CI = 1.109–4.223) but not of neck/lower back/right shoulder discomfort (*p* = 0.054), whereas rest breaks were a statistically insignificant predictor of both models. The arrangement of ‘sometimes/often’ with regular meal breaks compared to that of ‘never/rarely’ with regular meal breaks was significantly associated with a high risk of MSD and an OR greater than one. Homeworkers who ‘always’ arranged regular meal breaks showed an insignificant association with reduced MSD or neck/lower back/right shoulder discomfort. The arrangement of regular rest breaks was statistically insignificant to the risk of developing MSD (*p* = 0.157) and neck/lower/right shoulder (*p* = 0.172) discomfort.

#### 3.6.3. Home Workstation Setups

##### MSD

Logistic regression was performed to determine the effects of the chair and display screen on the probability that participants would have MSD (Table 8). 

The type of chair homeworkers used—that is, chair with a backrest and chair without a backrest or sofa, lower chair, and bar stool—was a statistically significant predictor of MSD (*p* = 0.013, OR = 2.635, 95% CI = 1.228–5.655). Furthermore, the type of display screen—that is, desktop monitor(s) and laptop monitor only or desktop monitor only, was a statistically significant predictor of MSD (*p* = 0.012, OR = 2.818, 95% CI = 1.258–6.314). Logistic regression models were statistically significant for MSD (χ^2^(3) = 15.394, *p* < 0.001). The OR for both chair and display screen factors was greater than one, so homeworkers who used a laptop monitor as a display screen and a chair without a backrest/the sofa or a high/low chair were 2.635 and 2.828 times more likely to suffer from MSD, respectively.

##### Neck/Lower Back/Upper Back/Right Shoulder

Logistic regression was performed to determine the effects of the chair and display screen on the probability that participants would develop neck, lower back, upper back, and right shoulder discomfort (Table 9). The type of chair was a statistically insignificant predictor of the neck, lower back, upper back, and right shoulder discomfort. However, the type of display screen use—that is, laptop monitor or desktop monitor—was a statistically significant predictor of suffering from the neck (*p* = 0.012), lower back (*p* = 0.031), and upper back (*p* = 0.013) discomfort, but not of right shoulder (*p* = 0.124) discomfort. Homeworkers using a laptop monitor as a display screen were associated with an increased likelihood of suffering from the neck (OR = 3.033), lower back (OR = 2.689), and upper back (OR = 3.105) discomfort.

#### 3.6.4. Summary of Predictors of MSD

##### Demographic Characteristics and Work Arrangements

Gender in demographic characteristics and regular meal breaks in work arrangements were two predictors of MSD and neck/lower back/shoulder discomfort. However, the body height of homeworkers was not able to predict the risk of MSD. Females anticipated a higher risk of MSD than males. Furthermore, rest break arrangements were not able to predict the risk of MSD. The arrangement of ‘sometimes/often’ regular meal breaks could increase the risk of MSD (Table 10).

##### Home Workstation Setups

For home workstation setups, the display screen was a significant predictor of the risk of MSD and neck, lower back, and upper back discomfort; however, it was an insignificant predictor of right shoulder discomfort. For chair type in the home workstation setups, the only significant result was found for MSD prediction. A suitable display screen (e.g., a desktop screen instead of a laptop screen) and chair design (e.g., a chair with a backrest instead of a chair without a backrest and a high/low chair height) significantly predicted the risk of MSD (Table 11).

## 4. Discussion

### 4.1. Demographic Characteristics of Homeworkers and MSD

The findings indicated that there are gender differences in the risk of suffering from MSD. Female homeworkers reported a higher risk of MSD and neck/lower back/right shoulder discomfort than male homeworkers. Published scientific studies have found that women have a higher risk of musculoskeletal symptoms than men. The same results were obtained for office employees with prolonged computer use [55,56,57]. Shariat, Cardoso [58] reported a significant association between pain and gender, particularly on the left and right sides of one’s shoulders. The current study provided the same results for computer-based homeworkers.

Age was not a predictor of MSD in this study. A previous study found that the age of office workers was significantly associated with the intensity of shoulder pain [58]. Another study found that shoulder pain increased after age 50 for physically demanding jobs compared to sedentary ones [59]. 

The current study found an association between body height and neck/lower back/right shoulder in the chi-square test. Body height and musculoskeletal condition may have an association with the use of a fixed-height desk [60] or a low table, with a fixed-height dining chair, or with the low position of the display screen [61]. 

### 4.2. Work Arrangements of Homeworkers and MSD

The number of months of experience with WFH and the number of days of WFH per week had no significant association with self-reported MSD. Changes in total working hours during WFH and daily hours of computing work were also insignificant in this regard. These findings contradict those of previous studies on office work, and WFH homeworkers reported a higher risk of musculoskeletal risk for prolonged computer users [62,63,64,65,66].

Previous studies have shown that homeworkers have greater control over their work tasks, work schedules, office hours, and work-life balance because of the autonomous nature of working from home [17,67,68,69]. Homeworkers may perceive that they have higher control over their work schedules and may use their commute time for work. This could be the reason that homeworkers reported longer work hours while working at home and that a high percentage of homeworkers could perform prolonged computing work using a flexible work style without reporting MSD. These results did not show a correlation between computing work hours and musculoskeletal health.

This study also showed that regular meal breaks are a predictor of MSD. However, there was no prediction effect of rest breaks on MSD for homeworkers. Surprisingly, ‘sometimes/often’ regular meal breaks compared to ‘never/rare’ regular meal breaks predicted a higher risk of MSD. 

When working in the office, a fixed lunch hour is usually provided, and office workers are expected to take advantage of it. In this study, 22.9% of homeworkers never or rarely arranged a ‘regular’ meal break, indicating that when workers work from home, they adopt a flexible work style. The study results revealed that homeworkers have the benefit of scheduling meal breaks on their own instead of having a fixed meal schedule or a fixed meal time. Thus, companies should consider allowing homeworkers to set their own times for meal breaks.

Approximately 40% of homeworkers did not schedule a rest break at all. Because of the nature of flexible work arrangements and of autonomy in work control for homeworkers, mini breaks can be arranged according to their wishes rather than scheduled intentionally. Furthermore, a comparison of the arrangement of rest breaks between office employees and homeworkers showed that, unlike office employees, homeworkers did not take regular rest breaks. However, the WFH study showed that increased risk of physical and psychological fatigue for those who failed to take rest breaks. Homeworkers who put in prolonged hours without taking breaks often disregarded the need for their internal recovery [70].

Although total work hours during WFH and prolonged hours with computers did not show an association with MSD, frequent breaks are still needed to prevent physical fatigue. These findings will be helpful to human resources managers and management when considering WFH arrangements. Health education of homeworkers on taking regular breaks and on techniques to maintain a good work-life balance are of significant value in regularising WFH employees’ behaviors. Adopting a flexible work style and allowing more control are the most suitable methods to achieve this for homeworkers [17]. 

### 4.3. Home Workstation Setups of Homeworkers and MSD

Many factors have been linked to ergonomic practices and the effects of telework arrangements [71]. Optimal home office settings are still under-addressed in previous research studies [72,73]. However, significant ergonomic problems have been discussed in regard to WFH [73,74]. 

#### 4.3.1. Home Workstation Setups for Homeworkers in Hong Kong

Workspace: Only 19% of homeworkers had a study room/reading room for WFH. About 25% of homeworkers worked in the bedroom. People in Hong Kong live in tiny spaces. The space per person is approximately 25% smaller than in Tokyo [13,30], and the living and dining areas are usually combined. It would be a challenge to mix work, meals, and sleep in the same space. When the dining table or bedroom is occupied for work, does this affect family life and sleep quality? Further investigation of homeworkers’ physical well-being and sleep quality may be needed.

Desk and chair: Among the survey participants, most conducted their work on a dining table (40.9%) and a dining chair (34.1%). In a study carried out in Japan (N = 4112), the dining table (18.6%) and dining chair (21.1%) were the second most used types of furniture for WFH. A work desk/PC desk (63.2%) and a backrest work chair (55.7%) were the most used. Homeworkers in Japan seem to have better workspaces and furniture arrangements than in Hong Kong. When comparing the prevalence rate of MSD, our study found that 50.4% of participants reported neck symptoms and 44% of lower back symptoms during WFH. Meanwhile, in a study in Japan, 72% of participants reported neck/shoulder pain, and 52% of the participants reported back pain. Both studies revealed the high rate of musculoskeletal problems among homeworkers using home furniture for work. 

The type of chair used was a predictor of MSD. Homeworkers who used a chair with a backrest compared to homeworkers who used a chair without a backrest, a sofa, a lower chair, or a bar stool faced a significantly lower risk of MSD. 

In the Code of Practice for Working with Display Screen Equipment published by the Hong Kong Labour Department [31], the checklist includes adjustment of seat height to suit the body size of the computer user and adjustment of backrest and tilt to provide adequate lumbar support. If homeworkers do not have better seating options, an inappropriate seating height will pose MSD risks. The employer has a legal responsibility to take the necessary follow-up actions to protect the occupational health of homeworkers [31,75]. 

Risk assessment is the first step to identifying and assessing risks to the occupational safety and health of homeworkers according to the workstation condition. The employer shall, so far as is reasonably practicable, ensure that home furniture and equipment are suitable for homeworkers. Employers are advised to develop internal reimbursement policies on home working setups and related expenses [76]. When considering the small home spaces in Hong Kong, a folding table and folding chair in appropriate heights with a soft cushion seat and back support are affordable options in a limited home space environment.

Display screen and use of laptop computer: The findings provided useful information to compare desktop and laptop screen use. The position of the laptop and poor working posture, such as slouching and looking downwards, are correlated with reported musculoskeletal disorders [77]. Gerding, Syck [78] reported that because the laptop is used most of the time in the office and always in the home office, a built-in laptop screen is also ‘always’ used (55.1%). Because of the design of the laptop and its lower screen position, it leads to awkward postures of the neck and back, contributing to a high chance of developing MSD in the upper body. This matches the results of the current study. The use of laptop monitors poses an approximately three times higher risk of suffering from neck and upper back discomfort and a 2.7 times higher risk of suffering from lower back discomfort. The discomfort of the neck, upper back, and lower back are intercorrelated with spinal and musculoskeletal problems.

Input devices: Half of the survey participants used a laptop keyboard and external mouse (50%) as input devices, and less than 30% of workers used an external keyboard and mouse during WFH. The laptop is convenient to use in a mobile environment because it comes with a keyboard and mouse, and about 20% of homeworkers did not use an external keyboard and mouse. A study on home office equipment reported that the laptop keyboard and external mouse are ‘always’ equipped. Although an external keyboard and mouse would be the most ergonomically preferred settings, respondents ‘never’ used them at home during COVID-19 [78]. The current study highlighted higher reported musculoskeletal problems in the right wrist and right shoulder than on the left side. This may be because the right hand is the dominant hand for mouse work. The use of the mouse posed a musculoskeletal risk to the neck and shoulder due to arm abduction [79]. Employers should reimburse employees for the purchase of an external keyboard and mouse to lower MSD risk. The use of a laptop riser or simply a box to position the laptop screen at an appropriate distance and height can also improve the overall workstation setup.

#### 4.3.2. Workstation Ergonomics

In summary, the use of different types of furniture, display screens, and input devices is relevant for computer users to adopt different working postures. A workstation is an assembly comprising the computer screen, chair, desk, printer, accessories, or other peripheral items [47]. Office ergonomics refers to the application of ergonomic principles in an office setting to promote the health and safety of computer users. It includes a focus on the selection and use of office furniture, the setup and evaluation of computer workstations, the prevention of musculoskeletal injuries due to prolonged computer use, training and education on healthy computer use, and occupational health management in the use of computers in the workplace [36,46]. Office ergonomics can be widely applied in the WFH setting for prolonged computer use.

### 4.4. Implications

This study provides scientific evidence for reviewing occupational health regulations in Hong Kong for protecting computer users during WFH. This review will protect office workers as WFH becomes a new norm. The government can refer to the laws and collective agreements of some countries in the European Union as a reference [10,14]. When developing company internal guidelines or policies, employers should consider the business operations, the local culture, and the local home environment and involve the stakeholders in discussions.

A less-than-ideal environment was found for most homeworkers in this study. Management should now understand the unfavorable conditions under which homeworkers work. The study sends a clear message to employers about providing the necessary support, such as providing suitable furniture, assessing the home workstation setups, and offering ergonomic training to workers. Regular communication between homeworkers and businesses on the WFH topic and staff surveys should be encouraged. Homeworkers working in a noisy home or homeworkers living with dependent family members require mutual understanding and schedule modification by the human resources manager [66,76].

Although the ergonomics program at the workstation was not evaluated to assess its usefulness for reducing the risk of MSD in homeworkers, the program is widely promoted for protecting employee health and well-being [36]. Companies are encouraged to consider a holistic approach to looking after their homeworkers so as to create a healthy workplace.

The study also gave clear results that inappropriate furniture and workstations pose the risk of developing musculoskeletal disorders. If their home condition is not suitable for WFH, employees should raise their concerns with their employers.

Finally, interior designers and product designers can play an important role in creating a suitable home environment, suitable furniture, and suitable computing equipment. A user-oriented design of furniture and equipment that considers living and working purposes would be of great help for homeworkers.

### 4.5. Future Study Directions

The current study focuses on the work arrangements and factors of home workstations associated with MSD. Further investigation of the practicality and cost-effectiveness of implementing the WFH ergonomics program will generate valuable findings for companies and homeworkers. For example, a cost-benefit study can be conducted on the correlation between the availability of suitable furniture and equipment, training and education of homeworkers and advice on work arrangements and breaks, and outcomes for homeworkers’ musculoskeletal health and their physical well-being. When a future study is conducted in more in-depth human research, the inclusion of an ethics committee will be considered.

## 5. Conclusions

The study offered useful information on home workstation setups, work arrangements, and musculoskeletal conditions of homeworkers during WFH in Hong Kong. Employers are suggested to arrange flexible work styles with suitable workspaces and breaks for homeworkers and to provide health education on WFH arrangements. Most homeworkers do not have a suitable workspace or furniture according to the assessment checklist in the code of practice. This study found significant factors for predicting higher risks of MSD when using unsuitable chairs and laptop screens. It also painted a clear picture of the deficiency of workspaces and the unergonomic setup of workstations. Employers should arrange home workstation risk assessments and provide the right furniture and equipment where applicable.

The limitations of the present study were identified. First, the self-reported MSD levels may not reflect pre-existing MSD conditions. Second, the possibility of aggravation of discomfort due to inappropriate workstation setups or equipment is unknown and difficult to measure objectively. 

## Figures and Tables

**Table 1:** The prevalence rate of work-related musculoskeletal discomfort of homeworkers among 14 body regions.

Body Regions (N = 232)	Neck	Upper Back	Lower Back	Left Shoulder	Right Shoulder	Left Upper Arm	Right Upper Arm
Without discomfort (%)	49.6	54.3	56.0	63.4	58.6	72.8	68.1
With discomfort (%)	50.4	45.7	44.0	36.6	41.4	27.2	31.9
Discomfort level (Mean)	2.31	1.83	1.97	1.48	1.87	0.85	1.18
Discomfort Level (SD)	2.793	2.532	2.725	2.428	2.765	1.782	2.202
**Body Regions**	**Eyes**	**Left elbow**	**Right elbow**	**Left wrist**	**Right wrist**	**Left lower leg**	**Right lower leg**
Without discomfort (%)	57.3	78.0	76.3	78.4	69.4	78.4	77.2
With discomfort (%)	42.7	22.0	23.7	21.6	30.6	21.6	22.8
Discomfort level (Mean)	1.79	0.65	0.77	0.66	1.10	0.60	0.67
Discomfort level (SD)	2.403	1.53	1.717	1.58	2.063	1.426	1.581

**Table 2:** Discomfort frequency of the three most reported MSD body regions (neck, lower back, and right shoulder).

Discomfort Frequency (N = 232)	Neck		Lower Back		Right Shoulder	
	N	%	N	%	N	%
Never	67	28.9	91	39.2	92	39.7
Rarely (few times/month)	74	31.9	66	28.4	67	28.9
Frequently (few times/week)	64	27.6	47	20.3	47	20.3
Constantly (nearly every day)	27	11.6	28	12.1	26	11.2

Remark: The percentages may not add up to 100% due to rounding.

**Table 3:** Work arrangements of homeworkers.

Work Arrangements (N = 232)		N	Percentage (%)
No. of months WFH	Less than 1 month	39	16.8
	1 to 2 months	52	22.4
	2 to 3 months	80	34.5
	4 months or longer	61	26.3
No. of day WFH per week	1 day per week	24	10.3
	2 days per week	34	14.7
	3 days per week	51	22.0
	4 days per week	31	13.4
	5 days or more per week	92	39.7
Working hours during WFH (Work in office vs. WFH)	Increase by 1 to 2 h/day	47	20.3
	Increase by over 2 h/day	49	21.1
	Almost the same	96	41.4
	Decrease 1 to 2 h/day	20	8.6
	Decrease over 2 h/day	20	8.6
Hours with computing work	Less than 3 h	17	7.4
	3 to 6 h	31	13.4
	6 to 8 h	80	34.5
	8 h or more	104	44.8
Regular meal break	Never	15	6.5
	Rarely	38	16.4
	Sometimes	60	25.9
	Often	80	34.5
	Always	39	16.8
Regular rest break	No rest break is scheduled	89	38.4
	Every 30 min to 1 h	36	15.5
	Every 2 h	65	28.0
	Every 3 h	30	12.9
	Every 4 h	12	5.2

Remark: The percentages may not add up to 100% due to rounding.

**Table 4:** Home workstation setups for homeworkers.

Home Workstation Setups (N = 232)		N	Percentage (%)
Workspace	Living area	76	32.8
Dining area		52	22.4
Spare room/study room or reading area		44	19.0
Bedroom		57	24.6
Others		3	1.3
Work desk	Fixed height desk	87	37.5
Adjustable desk		9	3.9
Dining table		95	40.9
Folding table		19	8.2
Coffee table/low table/high table		16	6.9
Others		6	2.6
Chair	Swivel chair with height adjustable function	67	28.9
Swivel chair without height adjustable function		7	3.0
Dining chair		79	34.1
Fixed height and fixed back chair		22	9.5
Bench without back support		8	3.4
Folding chair with back support		9	3.9
Folding chair without back support		8	3.4
Sofa/armchair		18	7.8
Low chair/kid sized chair/bar stool		3	1.3
On the floor/on the bed		10	4.3
Others		1	0.4
Display screen	Laptop screen only	147	63.4
One desktop monitor only		23	9.9
Use two or more desktop monitors		7	3.0
Laptop screen as main and desktop monitor as supplementary use		17	7.3
Desktop monitor as main monitor and laptop screen as supplementary use		31	13.4
Two or more desktop monitors and laptop		4	1.7
Two or more laptop screens		3	1.3
Input devices	External keyboard and mouse	65	28.0
Laptop keyboard and external mouse		116	50.0
Laptop keyboard and mouse touch pad		45	19.4
Touch screen/stylus		6	2.6

Remark: The percentages may not add up to 100% due to rounding.

**Table 5:** Summarised results of demographic characteristics and work arrangement factors associated with musculoskeletal discomfort.

Factors (N = 232)	Any of All Body Regions Without Discomfort (N = 90, 38.8%) With Discomfort (N = 142, 61.2%)	Neck/Lower Back/Right Shoulder Without Discomfort (N = 98, 42.2%) With Discomfort (N = 134, 57.8%)
Demographic characteristics		
Gender	0.001 ***	<0.001 ***
Age	^ NS	NS
Height	NS	0.029 *
Work arrangements		
No. of months WFH	NS	NS
No. of day WFH per week	NS	NS
Working hours during WFH	NS	NS
Hours with computing work	NS	NS
Regular meal break	0.014 *	0.036 *
Regular rest break	NS	NS

Remarks: ^ NS: not significant. * *p* < 0.05 *** *p* < 0.001.

**Table 6:** Summarised results of home workstation setup factors associated with self-reported musculoskeletal discomfort.

Home Workstation Setups Factors (N = 232)	Any of All Body Regions	Neck	Lower Back	Upper Back	Right Shoulder
Workspace	^ NS	NS	NS	NS	NS
Work desk	0.023 *	NS	NS	NS	NS
Chair	0.004 **	0.024 *	0.024 *	NS	0.033 *
Display screen	0.003 **	0.021 *	NS	0.008 **	NS
Input devices	NS	NS	NS	NS	NS

Remarks: ^ NS: not significant. * *p* < 0.05 ** *p* < 0.01.

**Table 7:** Summary of multivariate logistic regression analyses for demographic characteristics and work arrangements and self-reported musculoskeletal discomfort.

		Body Regions with Discomfort (Yes/No)					
(N = 232)		With/Without MSD			Neck/Lower Back/Right Shoulder		
		*p*-Value	Odds Ratio	95% CI (Lower to Upper Bound)	*p*-Value	Odds Ratio	95% CI (Lower to Upper Bound)
Demographic characteristics							
Gender							
	Male		1.00			1.00	
	Female	0.015 *	2.330	1.181–4.597	0.016 *	2.280	1.164–4.467
Body height							
	160 cm or below		1.00			1.00	
	161 cm or above	0.771	0.906	0.468–1.757	0.557	0.824	0.432–1.572
Work arrangements							
Regular meal break							
	Never/Rarely		1.00			1.00	
	Sometimes/Often	0.024 *	2.165	1.109–4.223	0.054	1.912	0.988–3.700
	Always	0.783	0.887	0.376–2.089	0.720	0.855	0.363–2.016
Rest break							
	No rest break is scheduled		1.00			1.00	
	Rest break is scheduled	0.157	0.658	0.368–1.176	0.172	0.672	0.381–1.185

* *p* < 0.05.

**Table 8:** Summary of multivariate logistic regression analyses for home workstation and self-reported musculoskeletal discomfort.

		Body Regions with Discomfort (Yes/No)		
Home Workstation Setups (N = 232)		With/Without MSD		
		*p*-Value	Odds Ratio	95% CI (Lower to Upper Bound)
Chair				
	Chair with backrest		1.00	
	Chair without backrest, sofa, lower chair & bar stool	0.013 *	2.635	1.228–5.655
Display screen				
	Desktop monitor(s)		1.00	
	Laptop monitor only or with desktop monitor	0.012 *	2.818	1.258–6.314

* *p* < 0.05.

**Table 9:** Summary of multivariate logistic regression analyses for home workstation setups and neck, lower back, upper back, and right shoulder musculoskeletal discomfort.

		Body Regions with Discomfort (Yes/No)					
Home Workstation Setups (N = 232)		Neck			Lower Back		
		*p*-Value	Odds Ratio	95% CI (Lower to Upper Bound)	*p*-Value	Odds Ratio	95% CI (Lower to Upper Bound)
Chair							
	Chair with backrest		1.00			1.00	
	Chair without backrest, sofa, lower chair & bar stool	0.231	1.494	0.775–2.878	0.108	1.703	0.890–3.260
Display screen							
	Desktop monitor(s)		1.00			1.00	
	Laptop monitor only or with desktop monitor	0.012 *	3.033	1.282–7.177	0.031 *	2.689	1.096–6.593
		**Body Regions with Discomfort (Yes/No)**					
**Home Workstation Setups** **(N = 232)**		**Upper Back**			**Right Shoulder**		
		***p*** **-Value**	**Odds Ratio**	**95% CI** **(Lower to Upper Bound)**	***p*** **-Value**	**Odds Ratio**	**95% CI** **(Lower to Upper Bound)**
Chair							
	Chair with backrest		1.00			1.00	
	Chair without backrest, sofa, lower chair & bar stool	0.745	1.113	0.584–2.122	0.144	1.618	0.848–3.086
Display screen							
	Desktop monitor(s)		1.00			1.00	
	Laptop monitor only or with desktop monitor	0.013 *	3.105	1.268–7.604	0.124	1.967	0.830–4.663

* *p* < 0.05.

**Table 10:** Summarised results of demographic characteristics and work arrangements factors associated with musculoskeletal discomfort.

Predictors (N = 232)	Any of All Body Regions Without/with Discomfort	Neck/Lower Back/Right Shoulder Without/with Discomfort
Demographic characteristics		
Gender	0.015 *	0.016 *
Body height	^ NS	NS
Work arrangements		
Regular meal break	0.024 *	NS
Regular rest break	NS	NS

Remarks: ^ NS: not significant. * *p* < 0.05.

**Table 11:** Summarised results of home workstation setups factors associated with musculoskeletal discomfort.

	Without/with Discomfort				
+ Predictors (N = 232)	Any of All Body Regions	Neck	Lower Back	Upper Back	Right Shoulder
Chair	0.013 *	^ NS	NS	NS	NS
Display screen	0.012 *	0.012 *	0.031 *	0.013 *	NS

Remarks: + Work desk was removed from the model because all the regression results were insignificant. ^ NS: not significant. * *p* < 0.05.

## Data Availability

Not applicable.

## Appendix A

**Table A1:** Demographic characteristics of homeworkers.

Demographic Characteristics (N = 232)		N	Percentage (%)
Gender	Male	72	31.0
	Female	160	69.0
Age	18 to 24	2	0.9
	25 to 34	38	16.4
	35 to 44	77	33.2
	45 to 54	91	39.2
	55 to 64	24	10.3
Body height	Below 155 cm	36	15.5
	156 to 160 cm	58	25.0
	161 to 165 cm	50	21.6
	166 to 170 cm	37	15.9
	171 cm or above	51	22.0
Employment status	Full-time	218	94.0
	Part-time	10	4.3
	Self-employed	3	1.3
	Other	1	0.4
Employment position	Top management	27	11.6
	Middle management	70	30.2
	Supervisor	36	15.5
	General staff	75	32.3
	Professional	22	9.5
	Other	2	0.9

**Table A2:** The association between demographic characteristics and with/without musculoskeletal discomfort of any of all body regions.

Any of All Body Regions						
Demographic Characteristics (N = 232)	Without Discomfort	With Discomfort				
	N (%)	N (%)	*p*-Value	Χ^2^	df	Phi
Gender						
Male	39 (43.3%)	33 (23.2%)	0.001 ***	10.39	1	0.212
Female	51 (56.7%)	109 (76.8%)				
Age						
34 or below	17 (18.9%)	23 (16.2%)				
35 to 44	31 (34.4%)	46 (32.4%)	0.759	0.55	2	0.049
45 or above	42 (46.7%)	73 (51.4%)				
Body height						
160 cm or below	29 (32.6%)	64 (45.1%)	0.600	3.55	1	−0.124
161 cm or above	60 (67.4%)	78 (54.9%)				

Remarks: The percentages may not add up to 100% due to rounding; *** *p* < 0.001.

**Table A3:** The association between demographic characteristics and with/without neck or lower back or right shoulder discomfort.

Neck/Lower Back/Right Shoulder						
Demographic Characteristics (N = 232)	Without Discomfort	With Discomfort				
	N (%)	N (%)	*p*-Value	Χ^2^	df	Phi
Gender						
Male	42 (42.9%)	30 (22.4%)	<0.001 ***	11.081	1	0.219
Female	56 (57.1%)	104 (77.6%)				
Age						
34 or below	18 (18.4%)	22 (16.4%)				
35 to 44	34 (34.7%)	43 (32.1%)	0.788	0.477	2	0.045
45 or above	46 (46.9%)	69 (51.5%)				
Body height						
160 cm or below	31 (32%)	62 (46.3%)	0.029 *	4.791	1	−1.440
161 cm or above	66 (68%)	71 (53.7%)				

Remarks: The percentages may not add up to 100% due to rounding; * *p* < 0.05 *** *p* < 0.001.

**Table A4:** The association between work arrangements and with/without musculoskeletal discomfort of any of all body regions.

Any of All Body Regions						
Work Arrangements (N = 232)	Without Discomfort	With Discomfort				
	N (%)	N (%)	*p*-Value	Χ^2^	df	Phi
No. of WFH months						
Less than two months	32 (35.6%)	59 (41.5%)				
Two to three months	33 (36.7%)	47 (33.1%)	0.66	0.831	2	0.06
Three months or longer	25 (27.8%)	36 (25.4%)				
No. of WFH days per week						
1 to 2 days per week	20 (22.2%)	38 (26.8%)				
3 to 4 days per week	34 (37.8%)	48 (33.8%)	0.703	0.704	2	0.703
5 Days or above per week	36 (40.0%)	56 (39.4%)				
Working hours during WFH (Work in office vs. WFH)						
Increase working hours	38 (42.2%)	58 (40.8%)				
Almost the same working hours	37 (41.1%)	59 (41.5%)	0.972	0.056	2	0.016
Decrease working hours	15 (16.7%)	25 (17.6%)				
Hours of computing work						
Less than six hours per day	13 (14.4%)	30 (21.1%)	0.405	1.81	2	0.088
Six to eight hours per day	34 (37.8%)	46 (32.4%)				
Over eight hours per day	43 (47.8%)	66 (46.5%)				
Regular meal break						
Never/Rarely	25 (27.8%)	28 (19.7%)	0.014 *	8.486	2	0.191
Sometimes/often	44 (48.9%)	96 (67.6%)				
Always	21 (23.3%)	18 (12.7%)				
Regular rest break						
No rest is scheduled	30 (33.3%)	59 (41.5%)	0.21	1.573	1	−0.082
Rest break is scheduled	60 (66.7%)	83 (58.5%)				

Remarks: The percentages may not add up to 100% due to rounding; * *p* < 0.05.

**Table A5:** The association between work arrangements and with/without neck or lower back or right shoulder.

Neck/Lower Back/Right Shoulder						
Work Arrangements (N = 232)	Without Discomfort	With Discomfort	N = 232			
	N (%)	N (%)	*p*-Value	Χ^2^	df	Phi
No. of WFH months						
Less than two months	34 (34.7%)	57 (42.5%)				
Two to three months	39 (39.8%)	41 (30.6%)	0.314	2.316	2	0.10
Three months or longer	25 (25.5%)	36 (26.9%)				
No. of WFH days per week						
1 to 2 days per week	23 (23.5%)	35 (26.1%)				
3 to 4 days per week	37 (37.8%)	45 (33.6%)	0.79	0.471	2	0.045
5 or above per week	38 (38.8%)	54 (40.3%)				
Working hours during WFH (Work in office vs. WFH)						
Increase working hours	43 (43.9%)	53 (39.6%)				
Almost the same working hours	39 (39.8%)	57 (42.5%)	0.802	0.044	2	0.044
Decrease working hours	16 (16.3%)	24 (17.9%)				
Hours of computing work						
Less than six hours per day	15 (15.3%)	28 (20.9%)	0.477	1.481	2	0.08
Six to eight hours per day	37 (37.8%)	43 (32.1%)				
Over eight hours per day	46 (46.9%)	63 (47.0%)				
Regular meal break						
Never/Rarely	26 (26.5%)	27 (20.1%)	0.036 *	6.663	2	0.169
Sometimes/often	50 (51.0%)	90 (67.2%)				
Always	22 (22.4%)	17 (12.7%)				
Regular rest break						
No rest is scheduled	33 (33.7%)	56 (41.8%)	0.209	1.577	1	−0.082
Rest break is scheduled	65 (66.3%)	78 (58.2%)				

Remarks: The percentages may not add up to 100% due to rounding; * *p* < 0.05.

**Table A6:** The association between the home workstation setups and with/without musculoskeletal discomfort of any of all body regions.

Home Workstation Setups (N = 232)	Any of All Body Regions					
	Without Discomfort	With Discomfort				
	N (%)	N (%)	*p*-Value	Χ^2^	df	Phi
Workspace						
Living area/dining area	51 (56.7%)	77 (54.2%)				
Spare room/study room/reading area	21 (23.3%)	23 (16.2%)	0.174	3.492	2	0.123
Bedroom or other	18 (20.0%)	42 (29.6%)				
Work desk						
Fixed height/adjustable height	45 (50.0%)	51 (35.9%)				
Dining table	36 (40.0%)	59 (41.5%)	0.023 *	7.571	2	0.181
Folding table/coffee table/low/high desk	9 (10.0%)	32 (22.5%)				
Chair						
Chair with backrest (Swivel office chair, dining chair and folding chair)	80 (88.9%)	105 (73.2%)	0.004 **	8.222	1	0.188
Chair without backrest, sofa, low chair & bar stool	10 (11.1%)	38 (26.8%)				
Display screen						
Desktop only	19 (21.1%)	11 (7.7%)	0.003 **	8.739	1	0.194
Laptop monitor only/Desktop and laptop	71 (78.9%)	131 (92.3%)				
Input devices						
External keyboard and mouse	30 (33.3%)	35 (24.6%)				
Laptop keyboard and external mouse	47 (52.2%)	69 (48.6%)	0.066	5.43	2	0.153
Laptop keyboard and touch pad/touch screen/stylus	13 (14.4%)	38 (26.8%)				

Remarks: The percentages may not add up to 100% due to rounding; * *p* < 0.05 ** *p* < 0.01.

**Table A7:** The association between the home workstation setups and with/without neck, lower back, upper back, and right shoulder discomfort.

Home Workstation Setups (N = 232)	Neck						Lower Back					
	Without Discomfort	With Discomfort					Without Discomfort	With Discomfort				
	N (%)	N (%)	*p*-Value	Χ^2^	df	Phi	N (%)	N (%)	*p*-Value	Χ^2^	df	Phi
Workspace												
Living area/dining area	84 (58.3%)	41 (49.4%)					87 (53.7%)	38 (58.5%)				
Spare room/study room/reading area	28 (19.4%)	15 (18.1%)	0.227	2.968	2	0.114	36 (22.2%)	7 (10.8%)	0.122	4.203	2	0.136
Bedroom or other	32 (22.2%)	27 (32.5%)					39 (24.1%)	20 (30.8%)				
Work desk												
Fixed height/adjustable height	62 (43.1%)	33 (39.8%)					71 (43.0%)	24 (38.7%)				
Dining table	62 (41.3%)	32 (38.6%)	0.316	2.307	2	0.316	66 (40.0%)	28 (45.2%)	0.775	0.51	2	0.047
Folding table/coffee table/low/high desk	20 (13.9%)	18 (21.7%)					28 (17.0%)	10 (16.1%)				
Chair												
Chair with backrest (Swivel office chair, dining chair, folding chair)	122 (84.7%)	60 (72.3%)	0.024 *	5.121	1	0.15	136 (84.0%)	46 (70.8%)	0.024 *	5.071	1	0.149
Chair without backrest, sofa, low chair & bar stool	22 (15.3%)	23 (27.7%)					26 (16.0%)	29.2%)				
Display screen												
Desktop only	24 (16.7%)	5 (6.0%)	0.021 *	5.352	1	0.154	24 (14.8%)	5 (7.7%)	0.146	2.112	1	0.096
Laptop monitor only/Desktop and laptop	120 (83.3%)	78 (94%)					138 (85.2%)	60 (92.3%)				
Input devices												
External keyboard and mouse	47 (32.6%)	18 (21.7%)	0.106	4.497	2	0.141	48 (29.6%)	17 (26.2%)				
Laptop keyboard and external mouse	71 (49.3%)	42 (50.6%)					77 (47.5%)	36 (55.4%)	0.554	1.182	2	0.072
Laptop keyboard and touch pad/touch screen/stylus	26 (18.1%)	23 (27.7%)					37 (22.8%)	12 (18.5%)				
**Home Workstation Setups** **(N = 232)**	**Upper Back**						**Right Shoulder**					
	**Without Discomfort**	**With Discomfort**					**Without Discomfort**	**With Discomfort**				
	**N (%)**	**N (%)**	***p*** **-Value**	**Χ^2^**	**df**	**Phi**	**N (%)**	**N (%)**	***p*** **-Value**	**Χ^2^**	**df**	**Phi**
Workspace												
Living area/dining area	93 (56.4%)	32 (51.6%)					94 (57%)	31 (50%)				
Spare room/study room/reading area	31 (18.8%)	12 (9.4%)	0.780	0.496	2	0.047	33 (20%)	10 (16.1%)	0.248	2.792	2	0.111
Bedroom or other	41 (24.8%)	18 (29.0%)					38 (23%)	21 (33.9%)				
Work desk												
Fixed height/adjustable height	71 (43.0%)	24 (38.7%)					69 (41.8%)	26 (41.9%)				
Dining table	66 (40.0%)	28 (45.2%)	0.775	0.510	2	0.047	69 (41.8%)	25 (40.3%)	0.963	0.76	2	0.018
Folding table/coffee table/low/high desk	28 (17%)	10 (16.1%)					27 (16.4%)	11 (17.7%)				
Chair												
Chair with backrest (Swivel office chair, dining chair, folding chair)	134 (81.2%)	48 (77.4%)	0.523	0.408	1	0.042	138 (83.6%)	44 (71%)	0.033 *	4.551	1	0.142
Chair without backrest, sofa, low chair & bar stool	31 (18.8%)	14 (22.6%)					27 (16.4%)	18 (29%)				
Display screen												
Desktop only	27 (16.4%)	2 (3.2%)	0.008 **	6.980	1	0.175	24 (14.5%)	5 (8.1%)				
Laptop monitor only/Desktop and laptop	138 (83.6%)	60 (96.8%)					141 (85.5%)	57 (91.9%)	0.192	1.699	1	0.087
Input devices												
External keyboard and mouse	51 (30.9%)	14 (22.6%)					49 (29.7%)	16 (25.8%)				
Laptop keyboard and external mouse	79 (47.9%)	34 (54.8%)	0.456	1.569	2	0.083	80 (48.5%)	33 (53.2%)	0.796	0.457	2	0.045
Laptop keyboard and touch pad/touch screen/stylus	35 (21.2%)	14 (22.6%)					36 (21.8%)	13 (21.0%)				

Remarks: The percentages may not add up to 100% due to rounding; * *p* < 0.05 ** *p* < 0.01.

## References

[1] Dyer C., Shepherd K. (2021). Remote Work: Redesign Processes, Practices and Strategies to Engage a Remote Workforce.

[2] Nilles J. (1975). Telecommunications and organizational decentralization. IEEE Trans. Commun..

[3] Green N., Tappin D., Bentley T. (2020). Working from home before, during and after the COVID-19 pandemic: Implications for workers and organisations. N. Z. J. Employ. Relat..

[4] Sullivan C. (2003). What’s in a name? Definitions and conceptualisations of teleworking and homeworking. New Technol. Work. Employ..

[5] Biron M., Van Veldhoven M. (2016). When control becomes a liability rather than an asset: Comparing home and office days among part-time teleworkers. J. Organ. Behav..

[6] Shamir B., Salomon I. (1985). Work-at-home and the quality of working life. Acad. Manag. Rev..

[7] Bailey D.E., Kurland N.B. (2002). A review of telework research: Findings, new directions, and lessons for the study of modern work. J. Organ. Behav..

[8] Hill E.J., Miller B.C., Weiner S.P., Colihan J. (1998). Influences of the virtual office on aspects of work and work/life balance. Pers. Psychol..

[9] Egan B. Feasibility and cost benefit analysis. Proceedings of the International Telework Association Annual International Conference.

[10] Hynes M. (2014). Telework isn’t working: A policy review. Econ. Soc. Rev..

[11] Chu A.M.Y., Chan T.W.C., So M.K.P. (2022). Learning from work-from-home issues during the COVID-19 pandemic: Balance speaks louder than words. PLoS ONE.

[12] Derks D., Bakker A.B., Peters P., van Wingerden P. (2016). Work-related smartphone use, work–family conflict and family role performance: The role of segmentation preference. Hum. Relat..

[13] Vyas L., Butakhieo N. (2021). The impact of working from home during COVID-19 on work and life domains: An exploratory study on Hong Kong. Policy Des. Pract..

[14] Welz C., Wolf F. (2010). Telework in the European Union.

[15] Ferrara B., Pansini M., De Vincenzi C., Buonomo I., Benevene P. (2022). Investigating the Role of Remote Working on Employees’ Performance and Well-Being: An Evidence-Based Systematic Review. Int. J. Environ. Res. Public Health.

[16] Natomi K., Kato H., Matsushita D. (2022). Work-Related Stress of Work from Home with Housemates Based on Residential Types. Int. J. Environ. Res. Public Health.

[17] Wöhrmann A.M., Ebner C. (2021). Understanding the bright side and the dark side of telework: An empirical analysis of working conditions and psychosomatic health complaints. NewTechnol. Work. Employ..

[18] Debono M., Garzia C. (2023). Trade Union Members’ Experiences and Attitudes towards Working from Home during the Pandemic. Sustainability.

[19] Darouei M., Pluut H. (2021). Work from home today for a better tomorrow!. How working from home influences work-family conflict and employees’ start of the next workday. Stress Health.

[20] Davidescu A., Apostu S.-A., Paul A., Casuneanu I. (2020). Work flexibility, job satisfaction, and job performance among Romanian employees—Implications for sustainable human resource management. Sustainability.

[21] Kazekami S. (2020). Mechanisms to improve labor productivity by performing telework. Telecommun. Policy.

[22] Delanoeije J., Verbruggen M. (2020). Between-person and within-person effects of telework: A quasi-field experiment. Eur. J. Work. Organ. Psychol..

[23] Song Y., Gao J. (2020). Does telework stress employees out? A study on working at home and subjective well-being for wage/salary workers. J. Happiness Stud..

[24] Kapoor V., Yadav J., Bajpai L., Srivastava S. (2021). Perceived stress and psychological well-being of working mothers during COVID-19: A mediated moderated roles of teleworking and resilience. Empl. Relat. Int. J..

[25] Heiden M., Widar L., Wiitavaara B., Boman E. (2021). Telework in academia: Associations with health and well-being among staff. High. Educ..

[26] Giménez-Nadal J.I., Molina J.A., Velilla J. (2019). Work time and well-being for workers at home: Evidence from the American Time Use Survey. Int. J. Manpow..

[27] Zhao S.Z., Wong J.Y.H., Luk T.T., Wai A.K.C., Lam T.H., Wang M.P. (2020). Mental health crisis under COVID-19 pandemic in Hong Kong, China. Int. J. Infect. Dis..

[28] Leung L. (2004). Societal, organizational and individual factors in the adoption of telework. Impact and Issues in News Media: Toward Intelligent Societies.

[29] Chan S.M., Wong H., Chung R.Y., Au-Yeung T.C. (2021). Association of living density with anxiety and stress: A cross-sectional population study in Hong Kong. Health Soc. Care Community.

[30] Ng N. (2018). Hong Kong’s Small Flats ‘to Get Even Smaller’, Hitting Quality of Life. South China Morning Post.

[31] Labour Department (2003). Code of Practice for Working with Display Screen Equipment.

[32] Oha K., Animägi L., Pääsuke M., Coggon D., Merisalu E. (2014). Individual and work-related risk factors for musculoskeletal pain: A cross-sectional study among Estonian computer users. BMC Musculoskelet. Disord..

[33] Shabbir M., Rashid S., Umar B., Ahmad A., Ehsan S. (2016). Frequency of neck and shoulder pain and use of adjustable computer workstation among bankers. Pak. J. Med. Sci..

[34] Calik B.B., Yagci N., Oztop M., Caglar D. (2022). Effects of risk factors related to computer use on musculoskeletal pain in office workers. Int. J. Occup. Saf. Ergon..

[35] Chim J.M.Y., Chen T. (2021). Occupational Disease Compensation and Update on the Musculoskeletal Health of Office Employees in Hong Kong.

[36] Chim J.M.Y., Chen T. (2021). Implementation of an Office Ergonomics Program to Promote Musculoskeletal Health: A Case Study in Hong Kong. IISE Trans. Occup. Ergon. Hum. Factors.

[37] Burgess R.A., Thompson R.T., Rollman G.B. (2008). The effect of forearm posture on wrist flexion in computer workers with chronic upper extremity musculoskeletal disorders. BMC Musculoskelet. Disord..

[38] Szeto G.P.Y., Straker L., O’Sullivan P. (2009). Neck–shoulder muscle activity in general and task-specific resting postures of symptomatic computer users with chronic neck pain. Man. Ther..

[39] Ardahan M., Simsek H. (2016). Analyzing musculoskeletal system discomforts and risk factors in computer-using office workers. Pak. J. Med. Sci..

[40] Cheung J.P.Y., Fung B., Ip W.Y., Chow S.P. (2008). Occupational repetitive strain injuries in Hong Kong. Hong Kong Med. J..

[41] Chim J. (2014). Ergonomics for the Prevention of Musculoskeletal Disorders of Computer Users in Hong Kong, Singapore and Japan. J. Ergon. S.

[42] Colenberg S., Jylhä T., Arkesteijn M. (2021). The relationship between interior office space and employee health and well-being–a literature review. Build. Res. Inf..

[43] Robertson M.M., Ciriello V., Garabet A. (2013). Office ergonomics training and a sit-stand workstation: Effects on musculoskeletal and visual symptoms and performance of office workers. Appl. Ergon..

[44] Roossien C.C., Stegenga J., Hodselmans A.P., Spook S.M., Koolhaas W., Brouwer S., Verkerke G.J., Reneman M.F. (2017). Can a smart chair improve the sitting behavior of office workers?. Appl. Ergon..

[45] Van Niekerk S.-M., Louw Q., Hillier S. (2012). The effectiveness of a chair intervention in the workplace to reduce musculoskeletal symptoms. A systematic review. BMC Musculoskelet. Disord..

[46] Chim J.M. (2014). The FITS model office ergonomics program: A model for best practice. Work.

[47] Labour Department (2009). A Health Guide on Working in Display Screen Equipment.

[48] Labour Department (2010). A Guide to Work with Computers.

[49] Safe Work Australia (2020). How Do I Setup a Workstation at Home?.

[50] Safe Work Australia (1991). Ergonomic Principles and Checklists for the Selection of Office Furniture and Equipment.

[51] Shaikh A., Kadrekad S. (2020). Impact of work from home in COVID-19: A survey on musculoskeletal problems in it professionals. Int. J. Allied Med. Sci. Clin. Res..

[52] Marley R.J., Kumar N. (1996). An improved musculoskeletal discomfort assessment tool. Int. J. Ind. Ergon..

[53] Choi S.D. (2010). Ergonomic assessment of musculoskeletal discomfort of iron workers in highway construction. Work.

[54] Sperandei S. (2014). Understanding logistic regression analysis. Biochem. Med..

[55] Janwantanakul P., Pensri P., Jiamjarasrangsri V., Sinsongsook T. (2008). Prevalence of self-reported musculoskeletal symptoms among office workers. Occup. Med..

[56] Juul-Kristensen B., Søgaard K., Støyer J., Jensen C. (2004). Computer users’ risk factors for developing shoulder, elbow and back symptoms. Scand. J. Work. Environ. Health.

[57] Wahlström J. (2005). Ergonomics, musculoskeletal disorders and computer work. Occup. Med..

[58] Shariat A., Cardoso J.R., Cleland J.A., Danaee M., Ansari N.N., Kargarfard M., Tamrin S.B.M. (2018). Prevalence rate of neck, shoulder and lower back pain in association with age, body mass index and gender among Malaysian office workers. Work.

[59] Hodgetts C.J., Leboeuf-Yde C., Beynon A., Walker B.F. (2021). Shoulder pain prevalence by age and within occupational groups: A systematic review. Arch. Physiother..

[60] Shikdar A.A., Al-Kindi M.A. (2007). Office ergonomics: Deficiencies in computer workstation design. Int. J. Occup. Saf. Ergon..

[61] Straker L., Burgess-Limerick R., Pollock C., Murray K., Netto K., Coleman J., Skoss R. (2008). The impact of computer display height and desk design on 3D posture during information technology work by young adults. J. Electromyogr. Kinesiol..

[62] Montreuil S., Lippel K. (2003). Telework and occupational health: A Quebec empirical study and regulatory implications. Saf. Sci..

[63] Calik B.B., Yagci N., Gursoy S., Zencir M. (2014). Upper extremities and spinal musculoskeletal disorders and risk factors in students using computers. Pak. J. Med. Sci..

[64] Kaliniene G., Ustinaviciene R., Skemiene L., Vaiciulis V., Vasilavicius P. (2016). Associations between musculoskeletal pain and work-related factors among public service sector computer workers in Kaunas County, Lithuania. BMC Musculoskelet. Disord..

[65] Ijmker S., Huysmans M.A., Blatter B.M., Van Der Beek A.J., Van Mechelen W., Bongers P.M. (2007). Should office workers spend fewer hours at their computer? A systematic review of the literature. Occup. Environ. Med..

[66] Beckel J.L., Fisher G.G. (2022). Telework and worker health and well-being: A review and recommendations for research and practice. Int. J. Environ. Res. Public Health.

[67] Jamal M.T., Anwar I., Khan N.A., Saleem I. (2021). Work during COVID-19: Assessing the influence of job demands and resources on practical and psychological outcomes for employees. Asia-Pac. J. Bus. Adm..

[68] Tremblay D.-G., Thomsin L. (2012). Telework and mobile working: Analysis of its benefits and drawbacks. Int. J. Work. Innov..

[69] Thulin E., Vilhelmson B., Johansson M. (2019). New telework, time pressure, and time use control in everyday life. Sustainability.

[70] Cropley M., Weidenstedt L., Leick B., Sütterlin S. (2022). Working from home during lockdown: The association between rest breaks and well-being. Ergonomics.

[71] Black N.L., St-Onge S. (2022). Measuring pandemic home-work conditions to determine ergonomic recommendation relevance. Work.

[72] Carayon P., Smith M.J. (2000). Work organization and ergonomics. Appl. Ergon..

[73] de Macêdo T.A.M., Cabral E.L.D.S., Silva Castro W.R., de Souza Junior C.C., da Costa Junior J.F., Pedrosa F.M., da Silva A.B., de Medeiros V.R.F., de Souza R.P., Cabral M.A.L. (2020). Ergonomics and telework: A systematic review. Work.

[74] Bentley T., Teo S., McLeod L., Tan F., Bosua R., Gloet M. (2016). The role of organisational support in teleworker wellbeing: A socio-technical systems approach. Appl. Ergon..

[75] Labour Department (2020). A Brief on the Occupational Safety and Health (Display Screen Equipment) Regulation.

[76] International Labour Organization (2020). An Employers’ Guide on Working from Home in Response to the Outbreak of COVID-19.

[77] Tanzila R.A., Prameswarie T., Hartanti M.D., Denaneer T. (2021). The Correlation between Position and Duration Use of Laptops with Musculoskeletal Disorders (MSDs). Mutiara Med. J. Kedokt. Dan Kesehat..

[78] Gerding T., Syck M., Daniel D., Naylor J., Kotowski S.E., Gillespie G.L., Freeman A.M., Huston T.R., Davis K.G. (2021). An assessment of ergonomic issues in the home offices of university employees sent home due to the COVID-19 pandemic. Work.

[79] Cook C., Burgess-Limerick R., Chang S. (2000). The prevalence of neck and upper extremity musculoskeletal symptoms in computer mouse users. Int. J. Ind. Ergon..

